# Identification of autoantigens and their potential post-translational modification in EGPA and severe eosinophilic asthma

**DOI:** 10.3389/fimmu.2023.1164941

**Published:** 2023-06-02

**Authors:** Ilaria Esposito, Ioanna Kontra, Chiara Giacomassi, Sotiria Manou-Stathopoulou, James Brown, Richard Stratton, Galateia Verykokou, Roberto Buccafusca, Michael Stevens, Ahuva Nissim, Myles J. Lewis, Paul E. Pfeffer

**Affiliations:** ^1^ William Harvey Research Institute, Queen Mary University of London, London, United Kingdom; ^2^ Department of Rheumatology, Royal Free NHS Foundation Trust, London, United Kingdom; ^3^ Department of Respiratory Medicine, Royal Free NHS Foundation Trust, London, United Kingdom; ^4^ Centre for Rheumatology, University College London, London, United Kingdom; ^5^ Department of Respiratory Medicine, Barts Health NHS Trust, London, United Kingdom; ^6^ School of Physical and Chemical Sciences, Queen Mary University of London, London, United Kingdom; ^7^ Department of Clinical Immunology, Barts Health NHS Trust, London, United Kingdom; ^8^ Department of Clinical Immunology, University Hospitals Sussex NHS Foundation Trust, Brighton, United Kingdom

**Keywords:** eosinophil, autoantibodies, autoantigens, oxidative modification, diagnostic test, TREM1, eosinophil peroxidase, NETosis

## Abstract

**Background:**

The chronic airway inflammation in severe eosinophilic asthma (SEA) suggests potential autoimmune aetiology with unidentified autoantibodies analogous to myeloperoxidase (MPO) in ANCA-positive EGPA (eosinophilic granulomatosis with polyangiitis). Previous research has shown that oxidative post-translational modification (oxPTM) of proteins is an important mechanism by which autoantibody responses may escape immune tolerance. Autoantibodies to oxPTM autoantigens in SEA have not previously been studied.

**Methods:**

Patients with EGPA and SEA were recruited as well as healthy control participants. Autoantigen agnostic approach: Participant serum was incubated with slides of unstimulated and PMA-stimulated neutrophils and eosinophils, and autoantibodies to granulocytes were identified by immunofluorescence with anti-human IgG FITC antibody. Target autoantigen approach: Candidate proteins were identified from previous literature and FANTOM5 gene set analysis for eosinophil expressed proteins. Serum IgG autoantibodies to these proteins, in native and oxPTM form, were detected by indirect ELISA.

**Results:**

Immunofluorescence studies showed that serum from patients with known ANCA stained for IgG against neutrophils as expected. In addition, serum from 9 of 17 tested SEA patients stained for IgG to PMA-stimulated neutrophils undergoing NETosis. Immunofluorescent staining of eosinophil slides was evident with serum from all participants (healthy and with eosinophilic disease) with diffuse cytoplasmic staining except for one SEA individual in whom subtle nuclear staining was evident. FANTOM5 gene set analysis identified TREM1 (triggering receptor expressed on myeloid cells 1) and IL-1 receptor 2 (IL1R2) as eosinophil-specific targets to test for autoantibody responses in addition to MPO, eosinophil peroxidase (EPX), and Collagen-V identified from previous literature. Indirect ELISAs found high concentrations of serum autoantibodies to Collagen-V, MPO, and TREM1 in a higher proportion of SEA patients than healthy controls. High concentrations of serum autoantibodies to EPX were evident in serum from both healthy and SEA participants. The proportion of patients with positive autoantibody ELISAs was not increased when examining oxPTM compared to native proteins.

**Discussion:**

Although none of the target proteins studied showed high sensitivity for SEA, the high proportion of patients positive for at least one serum autoantibody shows the potential of more research on autoantibody serology to improve diagnostic testing for severe asthma.

**Clinical trial registration:**

ClinicalTrials.gov, identifier, NCT04671446.

## Introduction

The pivotal importance of eosinophils in the pathology of severe asthma and eosinophilic granulomatosis with polyangiitis (EGPA) is increasingly evident given the success of anti-eosinophil biologic therapies (1, 2). However, the persistent chronicity of the eosinophilic airway inflammation in the absence of a known chronic, exogenous stimulus in many patients is unexplained and suggests the presence of an endogenous stimulus of persistent auto-reactive airway inflammation —an auto-immune hypothesis for severe asthma pathology —which with molecular spread could then potentially lead to the systemic disease of EGPA (3).

Autoantibody responses have been reported in patients with asthma (3). Lott and colleagues detected autoantibodies to collagen in asthmatic individuals (4). Similarly, Liu and colleagues have reported autoantibodies to collagen in asthmatic patients, as well autoantibodies to other proteins such as Activin A receptor, with clinical correlates to markers of asthma severity (5). Anti-neutrophil cytoplasmic antibodies (ANCA), especially anti-myeloperoxidase (MPO) antibodies, are present in a proportion of patients with EGPA but absent in a similar proportion. Similar perinuclear ANCA is also present in inflammatory bowel disease, raising the question of whether ANCA in EGPA is an epiphenomenon or pathological. Interestingly, recent research has reported autoantibodies to eosinophil peroxidase (EPX), an enzyme closely related to myeloperoxidase, in severe asthma sputum (6–8). Interestingly, recent research has reported autoantibodies to eosinophil peroxidase (EPX), an enzyme closely related to myeloperoxidase, in severe asthma sputum. However, none of these autoantibodies yet reported are present in the serum at high prevalence in severe asthma.

Previous research at our centre and with collaborators has shown that post-translational modification of potential autoantigens is a key step in breaking self-tolerance and development of autoimmune disease (9, 10). We have shown that serum autoantibodies to oxidative post-translationally modified (oxPTM) type II collagen are more frequent than those to native type II collagen in rheumatoid arthritis (11). Similarly, in type 1 diabetes mellitus, autoantibodies to insulin that has undergone oxidative post-translational modification are significantly more common than those to native insulin (12). Oxidative stress, implicated in the formation of post-translationally modified autoantigens, is a major feature of uncontrolled asthma inflammation (13). However, to date, the potential role of modified autoantigens in the airways has not been studied in severe eosinophilic asthma, EGPA, and other related diseases.

Identification of prevalent autoantibodies in severe asthma, EGPA, and related diseases is important not only for understanding disease pathogenesis but also in terms of a potential diagnostic test. Diagnosis of asthma and severe asthma, including of patients needing biologic therapy, is often difficult with delays in many cases leading to a significant unmet need (14–16). In rheumatoid arthritis and other types of inflammatory arthritis, the discovery of serum autoantibodies to cyclic citrullinated protein (CCP) and other autoantigens has revolutionised disease diagnosis, facilitating early management (17, 18). The identification of similarly diagnostic, serum autoantibodies in severe asthma would greatly advance end-to-end pathway management of these patients.

In this research, we have looked for potential serum autoantibodies to relevant proteins both in native form and in oxPTM form, using antigen-agnostic and targeted approaches. In the agnostic approach, we aimed to detect autoantibodies to stimulated granulocytes, whereby cellular induction of reactive oxygen species (ROS) in neutrophil/eosinophil extracellular trap (NET/EET) formation can cause oxidation of granulocyte self-antigens (19, 20). In the targeted approach, we have investigated autoantibodies against specific proteins of interest from prior literature (collagen V, MPO, and EPX) and other proteins highly expressed in eosinophils as identified by FANTOM5 gene set analysis (FANTOM Consortium 2014). The success of eosinophil suppressing biologic therapies suggests that eosinophils are a prime candidate as a possible source of autoantigens in severe asthma in some individuals.

## Materials and methods

### Participant recruitment and sampling

Patients with severe eosinophilic asthma (severe asthma with/without nasal polyps), patients with EGPA, healthy control (HC) participants, and a group of patients with other similar diseases for comparison [patients with mild-to-moderate asthma, eosinophilic chronic obstructive pulmonary disease (COPD), and those with other vasculitides] were recruited with informed consent (NHS REC ethics approval 20/PR/0004). Severe eosinophilic asthma (SEA) patients had a diagnosis as per ERS/ATS criteria confirmed by specialist clinic multi-disciplinary consensus as per standard UK practice (21, 22), with a recorded blood eosinophil count of ≥0. 3 × 10^9^/L on inhaled corticosteroids. For ELISAs, these patients were subdivided based on the presence or absence of nasal polyps: as severe eosinophilic asthma with nasal polyps (SEA+NP) or without nasal polyps (SEA-NP). Patients with EGPA had a clinical diagnosis of such and were confirmed to meet the research criteria suggested by Wechsler and colleagues (23). Patients with chronic obstructive pulmonary disease (COPD) and those with granulomatosis with polyangiitis or ANCA-associated vasculitis other than EGPA (GPA/AAV) were diagnosed as such by their specialist clinical teams. HC s were required to have no acute medical illness; no diagnosed chronic respiratory disease, allergy, or infective condition, including no history of asthma; and no history of EGPA or other vasculitis; and to not be taking any systemic immunosuppressive medication. Rituximab, plasmapheresis, and/or polyclonal immunoglobulin infusion (ever) were ineligibility criteria for study participation. The recruited patient populations included both those with a new diagnosis prior to definitive treatment and those already established on definitive treatment such as a biologic or steroid-sparing immunosuppressant medication.

Clinical data for participants were extracted from electronic medical records by their clinical teams and used to confirm patient protocol eligibility and for phenotyping patients. Blood was collected in appropriate serum separator tubes, and serum was aliquoted and stored at −80°C pending use in assays. Owing to the COVID pandemic, there were delays in completing recruitment and some experimental assays had to be run before all participants were recruited (in particular anti-MPO and anti-Collagen V ELISAs; **Supplemental Table S1**).

### Neutrophil immunofluorescence

Venous blood from healthy human volunteers (separate from the study serum donors) was collected in EDTA vacutainer tubes (BD Biosciences) and neutrophils were isolated using the density gradient medium Polymorphprep (Proteogenix) as per the manufacturer’s instructions. Isolation of neutrophils was confirmed by flow cytometry using a PerCP/Cyanine5.5 anti-human CD11b antibody (BioLegend) and a PE/Cyanine7 anti-human CD16 antibody (BioLegend). Neutrophils were plated on microscope slides (Hendley-Essex) at a concentration of 2 × 10 ^6^ cells/ml (100 μl/well) and incubated for 30 min at 37°C with 5% CO_2_ to allow adherence. For NETosis slides, cells were stimulated with 100 μl of PMA (final concentration 400 nM) in HBSS buffer (Gibco) containing 2 mM CaCl_2_ (Sigma) for 4 h at 37°C with 5% CO_2_. Unstimulated neutrophils were incubated for 4 h in HBSS buffer, in parallel. Neutrophils were then fixed and permeabilised with 95% ethanol for 15 min at −20°C, washed with PBS, and incubated with a 1:20 dilution of serum from study participants in PBS for 20 min at room temperature. After washing, a 1:320 dilution of anti-human IgG FITC conjugate antibody (Dako) in PBS was applied to each well for 20 min at room temperature in the dark. DNA was stained with 4′,6-diamidino-2-phenylindole (DAPI) for 20 min at room temperature in the dark. The stained cells were imaged using an LSM800 Zeiss fluorescence microscope using the program ZenBlue. At ×20 optical magnification, three random fields were captured for each sample and the images were acquired. These images were then analysed using ImageJ software by a clinical immunologist experienced in reading clinical ANCA slides and blinded to the disease status of study participants. Exposure times of each channel (blue or green) were kept constant throughout the analysis.

### Eosinophil immunofluorescence

Whole blood from healthy human volunteers was collected in ACD vacutainer tubes (BD Biosciences) and layered onto Lymphoprep (STEMCELL Technologies) as per the manufacturer’s instructions and centrifuged at 2,000 rpm for 30 min at room temperature with break off. The plasma layer, mononuclear cell band, and the density gradient medium were discarded to leave the red blood cell/polymorphonuclear pellet. Lysis of red blood cells in the pellet was performed using ACK lysis solution (Sigma), 10 mM potassium bicarbonate, and 97.3 μM EDTA. Eosinophils were isolated from the total polymorphonuclear cells by magnetic selection using the EasySep Human Eosinophil Isolation Kit (STEMCELL Technologies) as per the manufacturer’s instructions. Isolation of eosinophils was confirmed by flow cytometry using a PerCP/Cyanine5.5 anti-human CD11b antibody (BioLegend) and a Pacific Blue anti-human CRTH2 antibody (BioLegend). Eosinophils were plated on microscope slides (Hendley-Essex) at a concentration of 2 × 10^6^ cells/ml (100 μl/well) and incubated for 4 h at 37°C with 5% CO_2_. Eosinophils were fixed and permeabilised with 95% ethanol for 15 min at −20°C, washed with PBS, and incubated with 0.1 M glycine for 10 min at room temperature. The cells were blocked using a serum-free Protein Block (Agilent Dako) for 30 min at room temperature before incubating with serum diluted 1:20 in Antibody Diluent (Agilent Dako) for 20 min at room temperature. After washing, a 1:320 dilution of anti-human IgG FITC conjugate antibody (Dako) was applied to each well for 20 min at room temperature in the dark, followed by DAPI staining. Eosinophil slides were then read as per neutrophil slides.

### Analysis of the FANTOM5 dataset for eosinophil-specific genes

RLE normalised FANTOM5 data (24) were downloaded from http://fantom.gsc.riken.jp/5/data/ and analysed as previously described (25). In brief, data were subsetted to include only unmanipulated and uncultured primary tissues (derived cells, stimulated cells, and cell lines were excluded) and restricted to NCBI gene transcripts. For each gene, the CAGE peak with the highest mean expression was used. Data were *Z* score normalised across all primary tissues and expression of each gene was ranked across all tissues. A specificity score was determined for all genes by counting the number of tissues showing increased gene expression *Z* score >3 (i.e., more than 3 SD above the mean expression across all tissues), so that the most tissue-specific genes would have the lowest specificity scores. After different cutoffs were tested for robustness, genes were considered specific to a tissue type using the following criteria: (i) the level of gene expression in that tissue was in the top three tissues (i.e., ranks 1–3); (ii) *Z* score >5 (i.e., >5 SD above the mean expression across all tissues); and (iii) specificity score <10 tissues. Gene modules for different cell types were consistent with lists of genes previously published by the FANTOM5 consortium for several cell types (26, 27).

### Target proteins and oxidation

Human recombinant myeloperoxidase (MPO; cat# BA1078, Origene), Interleukin-1 Receptor 2 (IL1R2; cat# 10111-HCCH, Sino Biological), Triggering Receptor Expressed On Myeloid Cells 1 protein (TREM1; cat# 10511-H08H, Sino Biological), Collagen V (ColV; cat# C3657, Sigma), full-length Eosinophil Peroxidase (EPX; cat# abx620092, Abbexa), and light-chain EPX (cat# abx653287, Abbexa) were sourced as “native” target antigens for indirect ELISAs. Oxidative post-translational modification of target proteins was also undertaken to produce oxPTM target antigens by incubation overnight at 37°C with sodium hypochlorite at respectively these concentrations: 0.1 mM, 1 mM, 0.5 mM, 0.5 mM, and 0.4 mM (oxPTM light-chain EPX not undertaken). Protein modifications were monitored by gradient 4%–20% reducing sodium dodecyl sulphate‐polyacrylamide gel electrophoresis (SDS-PAGE) followed by staining with Coomassie blue (Abcam). Protein modification with EPX was monitored by mass spectroscopy (**Supplemental Methods** and **Figures S1–S4**).

### Autoantibody indirect ELISAs

Polystyrene 96-well plates (Nunc MaxiSorp, Thermo Fisher) were coated with 500 ng/well of native/oxPTM proteins in 50 mM pH 9.6 sodium bicarbonate and incubated overnight at 4°C. Plates were washed and blocked with 2% (wt./vol.) dry milk powder (ChemCruz) in 1× 0.05% PBS-Tween-20 for 2 h at room temperature under agitation at 200 rpm. Human sera samples diluted 1:50 in 2% (wt./vol.) dry milk powder in PBS-Tween were added in duplicate (100 μl in each well), followed by 2 h incubation at room temperature.

Secondary goat anti-human IgG alkaline phosphatase (ALP) conjugate antibody (Jackson ImmunoResearch) diluted 1:5,000 or secondary goat anti-human IgG- horseradish peroxidase (HRP)-conjugated antibody (Jackson ImmunoResearch) diluted 1:1,000 in 2% (wt./vol.) dry milk powder in 1× PBS/Tween-20 0.05% was added to detect IL1R2, MPO, and EPX, or Collagen V and TREM1 proteins, respectively, and incubated for 1 h at room temperature avoiding light exposure.

HRP ELISAs were subsequently incubated with TMB reagent before being stopped with 1 M sulphuric acid and optical density (OD) for each sample read at 450 nm using a Fluostar Omega Plate reader (BMG LABTECH). ALP ELISAs were incubated with p-nitrophenyl phosphate reagent and optical density (OD) read at 405 nm.

### Statistics

The upper limit of normal (ULN) for ELISA ODs was set as 1.96 x SD above the mean for healthy participants. Statistics were analysed in R (version 4.1.2; www.r-project.org) as described in the text using the following additional packages: ggplot2, dplyr, cowplot, tabyl, janitor, and vcd. Statistical comparisons between two groups were performed using two-tailed unpaired Wilcoxon test and between three or more groups using Kruskal–Wallis test. *p*-values < 0.05 were considered significant.

## Results

### Participants

Patients with severe eosinophilic asthma (SEA) and those with EGPA were recruited from specialist clinics in London, UK, as was a comparison group of patients with other similar diseases, for example, patients with COPD, with granulomatosis with polyangiitis or ANCA-associated vasculitis other than EGPA (GPA/AAV). HC participants were recruited from the same geographical area.

Sixty-three patients with SEA were recruited, of whom 30 had nasal polyps. Seventeen patients with EGPA were recruited, of whom six were ANCA-positive when clinically tested at disease diagnosis and seven had nasal polyps. Ten patients were recruited to the comparison group with other similar diseases [one with moderate asthma (MA), four with GPA/AAV, and five with COPD]. Thirty HC participants were recruited. Participant characteristics are shown in **Table 1**.

**Table 1:** Participant characteristics.

Number of participants	Healthy Control	Severe Eosinophilic Asthma	EGPA	Other
	30	63	17	10 (MA = 1, GPA/AAV = 4, COPD = 5)
Sex				
** Female**	16	29	7	4
** Male**	14	34	10	6
**Age (mean)**	42	54.4	56.6	63.1
Smoking status				
** Never smoker**	25	36	11	4
** Ex-smoker**	2	26	4	3
** Current smoker**	0	1	0	2
Treatment status				
** On long-term oral corticosteroid**	N/A	19	14	3
** On biologic**	N/A	41	5	0
** On steroid-sparing immunosuppressant**	N/A	0	15	1

MA, moderate asthma. GPA/AAV, granulomatosis with polyangiitis or ANCA-associated vasculitis other than EGPA. COPD, chronic obstructive pulmonary disease. N/A, not applicable.

Numbers of participants except age (mean years). EGPA, eosinophilic granulomatosis with polyangiitis

### Granulocyte autoantibody immunofluorescence

To assess for serum autoantibodies against granulocyte, and in particular eosinophil, antigens in patients with SEA/EGPA and HCs in an unbiased, autoantigen-agnostic manner, immunofluorescence studies were conducted in the manner of clinical ANCA immunofluorescence tests.

**Figure 1:**
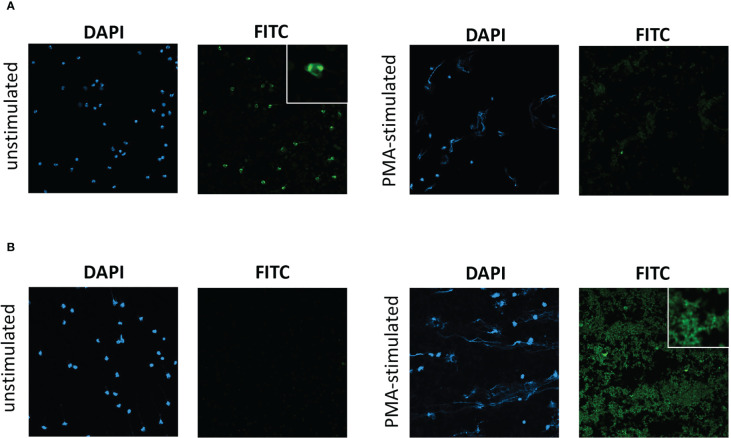
Immunofluorescent staining of serum IgG to unstimulated neutrophils and to PMA-stimulated neutrophils undergoing NETosis. Immunofluorescent staining with participant serum from **(A)** a patient with ANCA-positive EGPA, exhibiting IgG to unstimulated neutrophils but not to PMA-stimulated neutrophils, and from **(B)** a patient with severe eosinophilic asthma, exhibiting IgG to PMA-stimulated neutrophils but not unstimulated neutrophils. DAPI, 4′,6-diamidino-2-phenylindole staining. FITC, anti-human IgG FITC conjugate antibody staining. Photomicrographs of representative field of views with ×20 optical microscopy. Insets shows ×4 digital zoom images of characteristic features.

In initial experiments, slides of unstimulated neutrophils, and PMA-stimulated neutrophils with induced NETosis, from healthy donors separate from the HC participant group, were prepared. Slides were incubated with participant serum and then the presence of IgG in patient/HC serum to antigens on neutrophil slides was examined by immunofluorescence. Serum was tested from 29 participants [7 HC s, 17 patients with severe eosinophilic asthma (SEA) with/without nasal polyps, and 5 with EGPA; **Supplemental Table S2**]. For unstimulated neutrophil slides, strongly positive nuclear staining was evident with serum from two patients, one of whom had ANCA-positive (clinical lab test) EGPA (**Figure 1A**) and the other had severe eosinophilic asthma with positive ANCA (clinical lab test) but not meeting clinical criteria for EGPA. Weakly positive staining of borderline significance was evident with serum from three further patients (two with SEA and one with EGPA), with the remaining samples negative (see example in **Figure 1B**). For PMA-stimulated slides with evident NETosis, strongly positive IgG staining was evident with serum from 7 donors (5 severe eosinophilic asthma and 2 EGPA; **Figure 1B**), weakly positive staining was evident with serum from 5 participants (4 severe eosinophilic asthma and 1 HC), with the remaining 17 negative (see example in **Figure 1A**). The immunofluorescence from positive PMA-stimulated neutrophil slides was predominantly of clusters of speckles, not associated with cell nuclei. Of the two patients with nuclear staining of unstimulated neutrophils, one had weakly positive staining of the PMA-stimulated neutrophil slide and the other had no significant staining. Considering the 7 HC s as a group, none had a positive neutrophil immunofluorescence slide except one with a weakly positive staining for a PMA-stimulated slide. Considering the 17 SEA patients as a group (not including those with EGPA), 9 had a positive or weakly positive neutrophil immunofluorescence slide.

**Figure 2:**
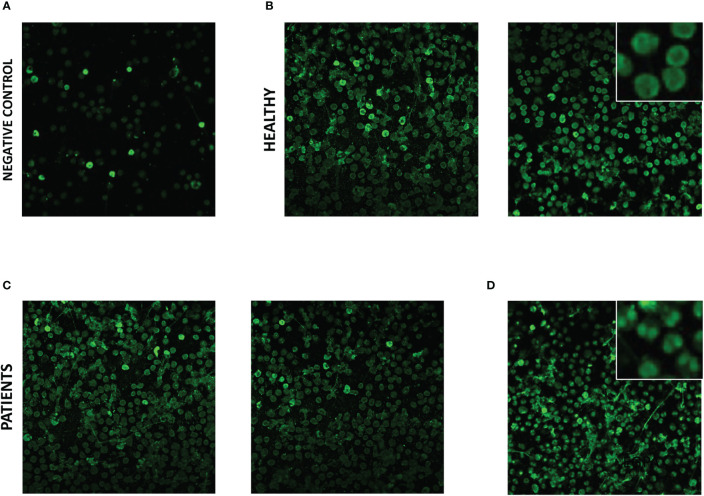
Immunofluorescent staining of serum IgG to eosinophils from healthy control participants and patients with severe eosinophilic asthma and EGPA. Eosinophil slide immunofluorescence with anti-human IgG FITC conjugate antibody staining; **(A)** in the absence of participant serum, **(B)** with serum from healthy control participants, **(C)** with serum from patients with ANCA-positive EGPA, and **(D)** with serum from ANCA-positive patient with severe eosinophilic asthma. Photomicrographs of representative field of views with ×20 optical microscopy. Insets shows ×4 digital zoom images of characteristic features.

In subsequent experiments, we aimed to examine eosinophil slides in a similar manner. However, use of an equivalent protocol for slide staining led to non-specific staining of eosinophils (positive immunofluorescence with anti-human IgG FITC conjugate antibody in the absence of addition of participant serum). Addition of a protein block to the protocol abrogated most of the non-specific staining (**Figure 2A**), and serum was tested from 11 participants [5 HC s, 2 patients with ANCA-positive EGPA, 1 with SEA, 1 with moderate asthma (MA), 1 with COPD, and 1 with GPA/AAV; **Supplemental Table S2**] using the adapted protocol. Diffuse cytoplasmic staining was evident with all eosinophil immunofluorescence slides, including those with serum from HC s (**Figure 2B**) and patients (**Figure 2C**), with subtle nuclear staining evident with serum from one patient with SEA known to be ANCA positive (**Figure 2D**). Given the non-specific immunofluorescence evident with HC eosinophil slides, and the subtle difference with slide exhibiting nuclear staining, the diagnostic use of staining for anti-eosinophil cytoplasmic antibodies (AECA) was not pragmatic and therefore attention shifted to a targeted protein approach.

### Indirect ELISA for serum autoantibodies to target proteins

**Figure 3:**
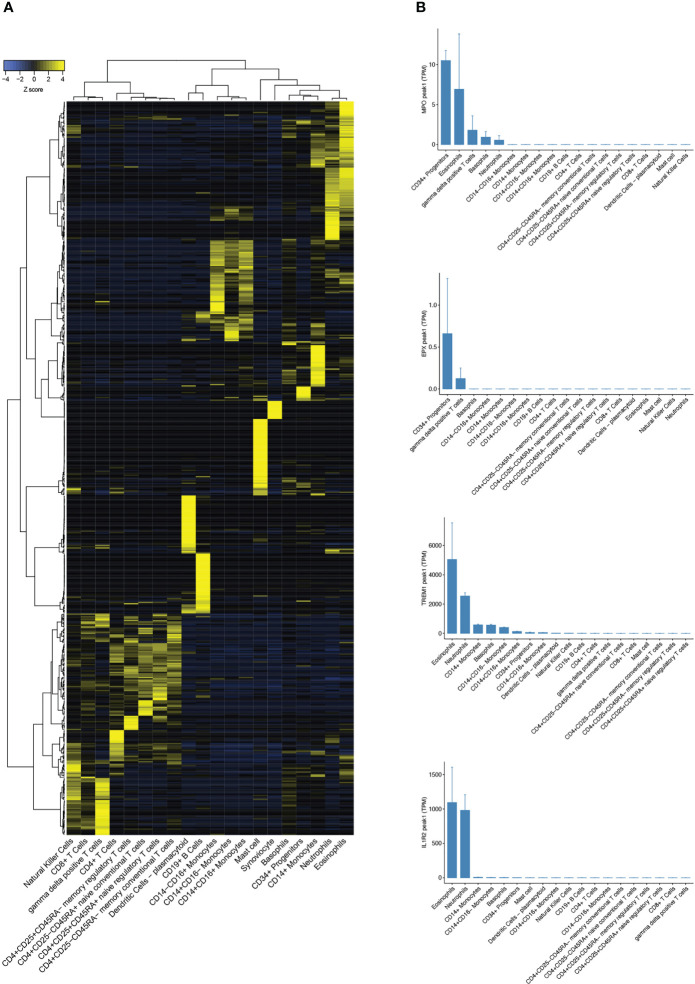
Analysis of FANTOM5 CAGE-sequencing tissue repository dataset. **(A)** Heatmap showing CAGE-Seq expression of cell-specific gene sets in peripheral blood subsets from FANTOM5. **(B)** Bar charts comparing transcript expression levels of eosinophil-specific genes *MPO*, *EPX*, *TREM1*, and *IL1R2* in human peripheral blood subsets.

Candidate target proteins for the indirect ELISA included those previously identified of interest in asthma, and other eosinophil highly expressed targets were identified by reanalysis of the FANTOM5 tissue repository gene set (**Figure 3A**) (24). Triggering Receptor Expressed On Myeloid Cells 1 protein (TREM1) and Interleukin-1 Receptor 2 (IL1R2) were identified as novel highly eosinophil-specific genes compared to other peripheral blood subset cell types (**Figure 3B**). We also compared these results with proteins considered to be granulocyte expressed including MPO and EPX. It was noteworthy that *MPO* transcript showed moderately specific expression in eosinophils with higher transcript levels compared to neutrophils. However, EPX, which is considered an eosinophil-specific protein, had negligible transcript levels in eosinophils and showed generally low expression in all cell types analysed. This is plausible because secreted proteins stored in granules, including numerous cytokines, may commonly demonstrate very low mRNA transcript levels once sufficient protein has been synthesised and stored in relevant cells within secretory granules.

**Figure 4:**
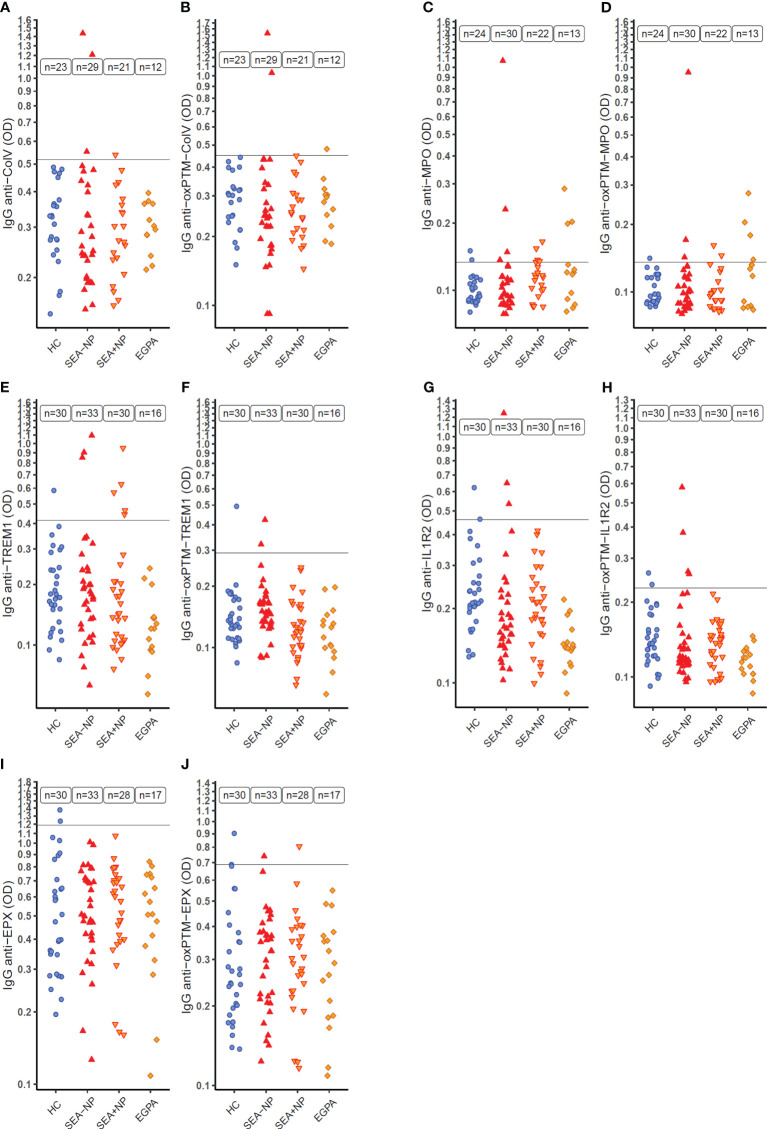
Serum autoantibodies to target native and oxidative post-translationally modified (oxPTM) proteins as measured by indirect ELISA. ELISA optical density (OD) on a logarithmic scale (*y*-axis) as a measure of serum IgG autoantibodies to target proteins in native and oxidative post-translationally modified form (oxPTM) in different participant groups (*x*-axis). **(A, B)** Collagen V (ColV); **(C, D)** Myeloperoxidase (MPO); **(E, F)** Triggering Receptor Expressed On Myeloid Cells 1 (TREM1); **(G, H)** Interleukin-1 Receptor 2 (IL1R2); **(I, J)** Eosinophil Peroxidase (EPX) HC, healthy control participants. SEA-NP, severe eosinophilic asthma without nasal polyps; SEA+NP, severe eosinophilic asthma with nasal polyps. EGPA, eosinophilic granulomatosis with polyangiitis. *y*-axis intercept at the upper limit of normal (ULN) for healthy control participant ELISA OD.

ELISA optical densities (ODs), as a measure of autoantibody serum concentrations, to Collagen V (ColV) above the Upper Limit of Normal (ULN, defined using ODs from HC s) —here after termed positive tests —were evident with serum from 4 SEA individuals (out of 50 tested; 8.0%) but none of the 23 HC s tested (**Figure 4A**, **Table 2**, **Supplemental Figure S5**). Serum samples positive for antibodies to oxPTM-ColV were evident in three individuals — two with SEA and one with EGPA, and no HC s (**Figure 4B**).

**Table 2:** Serum autoantibody positivity across participant groups.

Protein	HC	SEA (SEA-NP, SEA+NP)	EGPA
ColV	0.0%	8.0% (10.3%, 4.8%)	0.0%
oxPTM-ColV	0.0%	4.0% (6.9%, 0%)	8.3%
MPO	8.3%	17.3% (13.3%, 22.7%)	23.1%
oxPTM-MPO	4.2%	9.6% (10.0%, 9.1%)	30.8%
TREM1	3.3%	12.7% (9.1%, 16.7%)	0.0%
oxPTM-TREM1	3.3%	3.2% (6.1%, 0.0%)	0.0%
IL1R2	6.7%	4.8% (9.1%, 0.0%)	0.0%
oxPTM-IL1R2	6.7%	6.3% (12.1%, 0.0%)	0.0%
EPX	6.7%	0.0% (0.0%, 0.0%)	0.0%
oxPTM-EPX	6.7%	3.3% (3.0%, 3.6%)	0.0%

Percentage of participants by group with positive serum autoantibodies to each target protein as identified by indirect ELISA. Positive result, ELISA OD over the ULN as determined from the healthy control participant group. HC, healthy control; SEA, severe eosinophilic asthma; SEA-NP, severe eosinophilic asthma without nasal polyps; SEA+NP, severe eosinophilic asthma with nasal polyps; EGPA, eosinophilic granulomatosis with polyangiitis.

Nine of 52 participants (17.3%) with SEA had positive serum autoantibody status to unmodified MPO, and 3 of 13 participants (23.1%) with EGPA, compared to 2 of 24 (8.3%) HC s (**Figure 4C**). With oxPTM-MPO, proportions were similar; 5 of 52 with SEA (9.6%), 4 of 13 with EGPA (30.1%), and 1 of 24 HC s (4.2%) (**Figure 4D**).

Eight of 63 SEA patients tested (12.7%) had positive serum autoantibodies to TREM1 in contrast to 0 of 16 patients with EGPA (0.0%) and 1 of 30 HC s (3.3%) (**Figure 4E**). With oxPTM-TREM1, proportions were lower with 2 of 63 patients positive (3.2%) and 1 of 30 HC s (3.3%) (**Figure 4F**). Three of 63 SEA patients (4.8%) and 2 of 30 HC s (6.7%) had positive serum autoantibodies to IL1R2 (**Figure 4G**) with similar proportions positive for oxPTM-IL1R2 (**Figure 4H**).

In ELISAs for autoantibodies to EPX and oxPTM-EPX, ODs for both HC s and patients were notably higher than with other target protein ELISAs despite other aspects of the ELISA protocols being unchanged (**Figure 4I**). No patients had higher concentrations of serum autoantibodies to EPX than the ULN from the HC s. Results with oxPTM-EPX were similar (**Figure 4J**).

**Figure 5:**
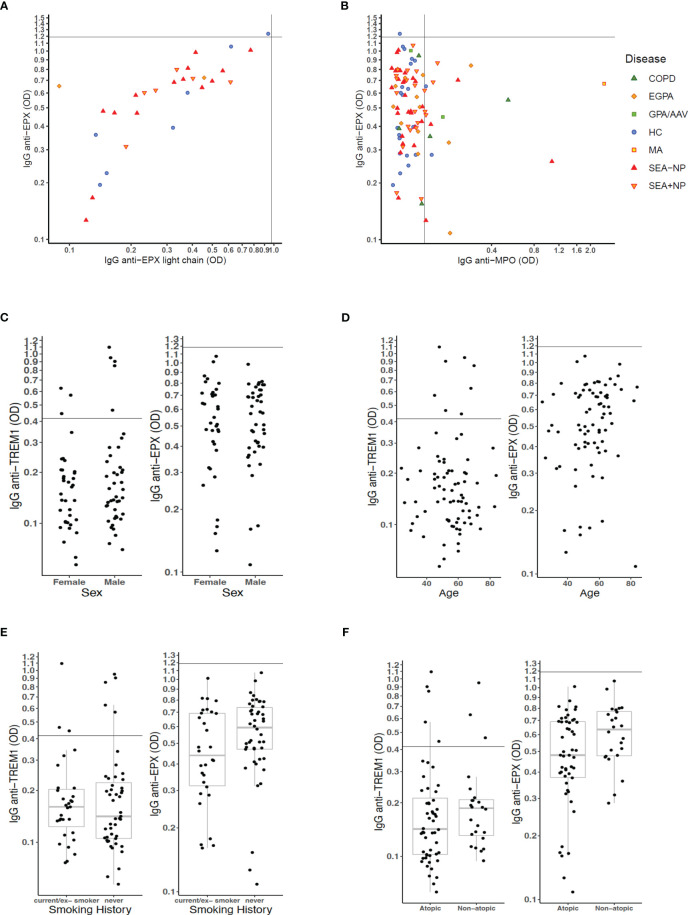
Analyses of serum autoantibody ELISA ODs relative to patient characteristics. **(A)** ELISA OD for serum IgG to full-length EPX protein compared to that for serum IgG to EPX light chain protein. **(B)** ELISA OD for serum IgG to full-length EPX protein compared to that for serum IgG to MPO protein. Scatter plot points for individual participants formatted as per **Figure 4** with axis–intercepts at upper limits of normal (ULN) for healthy control participants. **(C–F)** Effects of **(C)** sex, **(D)** age, **(E)** smoking status, and **(F)** atopic status on measures of serum IgG to TREM1 and EPX in patients with severe eosinophilic asthma and EGPA.

To examine whether the higher ODs with the EPX ELISAs might be due to non-specific reactions to the EPX protein used in the ELISA, in a small subset of patients, we conducted ELISAs using a different protein preparation of EPX (light chain only) and compared results. There was a strong positive correlation between ELISA ODs using the different EPX protein preparations (**Figure 5A**) consistent with the ELISAs measuring high concentrations of IgG to EPX in the serum of both HC s and patients, rather than non-specific signal. Comparing ELISA ODs to EPX and MPO, it was apparent that many patients with positive IgG to MPO also had high serum concentrations of IgG to EPX (**Figure 5B**).

In further analyses, we sought to understand within the group of patients with SEA and EGPA whether there were particular characteristics associated with higher concentrations (as measured by ELISA OD) for serum autoantibodies to TREM1 and EPX. There were no differences in concentrations by patient sex (**Figure 5C**) or patient age (**Figure 5D**). With regard to smoking status, the median anti-EPX OD was higher in never smokers than those with a smoking history but distribution was not significantly different (**Figure 5E**). However, non-atopic patients had significantly higher serum concentrations for anti-EPX IgG than atopic patients (Wilcoxon test, *p* = 0.034; **Figure 5F**). Whether or not the patient was on definitive treatment (with a biologic or steroid-sparing immunosuppressant) did not appear to have major impact on anti-TREM1 and anti-EPX serology, and neither did presence/absence of current blood eosinophilia (**Supplemental Figure S6**).

Of the nine severe asthma patients with positive immunofluorescence for PMA-stimulated neutrophil slides, six had a positive autoantibody ELISA, though there was no clear association with a specific autoantigen.

**Figure 6:**
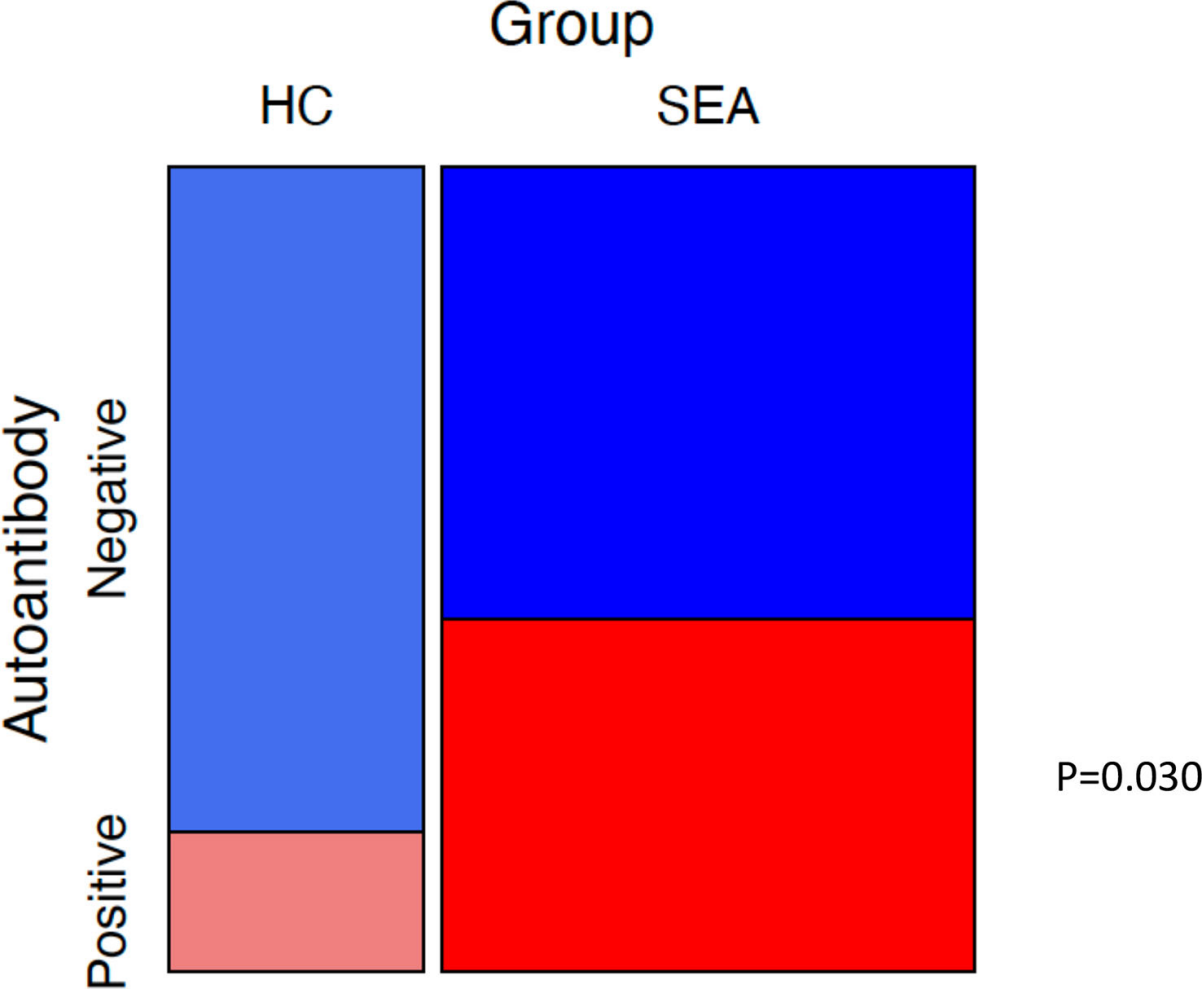
Mosaic plot of proportions of participants with positive autoantibody status. Mosaic plot of proportions of healthy control participants (HC) and severe eosinophilic asthma (SEA) patients negative or positive for serum autoantibodies to at least one of ColV, MPO, TREM1, or IL1R2. Positive defined as an ELISA OD above ULN. Boxes sized relative to patient number. *p*-value for chi-squared test.

Finally, we examined whether the proportion of participants positive to one or more of ColV, MPO, TREM1, and IL1R2 was higher in patients with SEA compared to HC s (**Figure 6**). Patients with EGPA were excluded from this analysis due to potential selection bias in that group in favour of ANCA-positive individuals. Of 46 SEA patients who had been tested against all four potential autoantigens, 19 were positive to at least one compared to 4 of 25 HC s (chi-squared test *p* = 0.030).

## Discussion

In this research, we set out to identify potential serum autoantibody responses in patients with severe eosinophilic asthma and EGPA using an approach agnostic to candidate autoantigens (granulocyte immunofluorescence) and a targeted approach (indirect ELISAs), and in particular to oxPTM autoantigens. In the agnostic approach, serum from a high proportion of severe eosinophilic asthma patients yielded IgG staining of PMA-stimulated neutrophils that had undergone NETosis in a manner indicative of serum antibodies against potentially oxidised products of NETosis. However, severe eosinophilic asthma is characterised by eosinophilic rather than neutrophilic inflammation and we therefore proceeded to look for serum antibodies to eosinophils using similar methodology. Nuclear eosinophil immunofluorescence was evident with serum from an ANCA-positive severe asthma patient but more notable was the diffuse cytoplasmic staining evident with all HC s and other patients. Given the aim of developing a clinical blood test to aid diagnosis of severe eosinophilic asthma and EGPA, and the subtlety of the difference in eosinophil staining pattern, we proceeded to the target protein approach. Indirect ELISAs found serum autoantibodies to ColV, MPO, and TREM1 in a higher proportion of SEA patients than HC s, and to IL1R2 in similar proportions. High titres of IgG to EPX were present in a high number of both patients and HC s. The proportion of SEA patients with autoantibodies positive to at least one of ColV, MPO, TREM1, and/or IL1R2 was significantly greater than the proportion of HC s, though no single autoantibody ELISA showed high sensitivity.

Whether elevated titres of autoantibodies to TREM1 in severe eosinophilic asthma, as identified in this research, are an epiphenomenon or whether these autoantibodies have a functional role in asthma pathology will require more research. TREM1 is a transmembrane protein expressed by immune cells with a functional role in amplifying certain immune responses. Interestingly, associations between asthma and TREM1 pathway signalling activity have been reported by several groups (28, 29), and relative suppression of the TREM1 signalling pathway has been reported in eosinophilic nasal polyposis (30, 31).

We hypothesised that serum autoantibodies in severe asthma and EGPA would include autoantibodies (to) to antigens post-translationally modified by oxidation in the airways. Oxidative burst is a feature of granulocyte degranulation and NETosis/EETosis, and the presence of antibodies in the serum of many patients with severe eosinophilic asthma to products of NETosis (may) could indicate antibodies (to) to oxidised neutrophil-derived proteins. However, none of the candidate protein autoantibodies were more prevalent to the oxidised than the native unmodified form. Although post-translational modification of proteins may not be a major mechanism in the development of auto-immune responses in the airway, protein oxidation is only one of many different types of post-translational modification that proteins may undergo and other types of PTM may be more important. For example, the action of eosinophil peroxidase has been linked to carbamylation of proteins (32). Histone citrullination is a feature of granulocyte extracellular trap formation and potentially other bystander proteins may also be citrullinated (33).

In this study, we have used stimulation of NET/EETosis as a physiological mechanism for generation of post-translationally modified granulocyte proteins. NETs and EETs have active functions *in vivo*. Of particular relevance to this research is their capacity to be immunogenic —NETs have been shown to facilitate uptake by dendritic cells of neutrophil antigens and thereby induction of ANCA autoantibodies (34). NETs may also be able to facilitate release of potential autoantigens by epithelial cells (35), an action that might be shared with EETs, which have been shown to similarly have effects on epithelial cells as well as activating other eosinophils (36). Products of NETosis can promote type 2 inflammation in murine models (37) and have recently been suggested as a potential biomarker in asthma (38).

However, the pathological role for autoantibodies may differ between anti- MPO-positive and ANCA-negative EGPA. Genomic research shows anti- MPO-positive EGPA to have a strong association at *HLA-DQ* consistent with autoantibody pathology whilst ANCA-negative EGPA has a separate but weaker association in the *HLA* region (39). Anti-PR3 ANCA-associated vasculitis has different genomic associations, particularly in the *HLA-DP* region (40, 41).

In our targeted autoantigen approach, we selected for assay proteins encoded by genes identified as being highly expressed with high specificity in eosinophils in the FANTOM5 dataset. The advantage of this approach was the ability to identify potential novel eosinophil-associated autoantigen proteins, in a dataset generated from primary cells rather than cell lines. We only investigated a proportion of the identified targets in this proof-of-principle study. A limitation of the FANTOM5 dataset is that it identifies gene transcripts rather than expressed proteins —not all transcripts are translated into proteins and the genes for some proteins are not continually expressed. An alternative would have been to use a protein dataset, and this should be considered in future research.

Our research indicates large-scale screening of different proteins and post-translational modifications may yet identify serum autoantibodies, alone or in combination as a panel, with good sensitivity and specificity for diagnosis of severe asthma and (ANCA-negative) EGPA.

It is important for any clinical test to have both good sensitivity for the disease but also high specificity. In this case, it would need good specificity for severe asthma, to differentiate from other causes of breathlessness and other eosinophilic conditions. Severe asthma is a heterogeneous condition and individual autoantibodies may show specificity for particular endotypes of severe asthma. To address this, and reflect real-world clinical conditions, we recruited a broad SEA patient group, without restricting patient characteristics further than being of an eosinophilic endotype. Clinical data were used to further characterise patients by the presence/absence of nasal polyps, atopy, and smoking history. COVID pandemic spirometry restrictions unfortunately prevented characterisation by the presence/absence of persistent airflow limitation. Our patient population was also heterogeneous in terms of disease stage, including both new patients who had recently completed initial assessment and those on definitive treatment with a biologic/steroid-sparing immunosuppressant —this was in keeping with our interest in a possible autoantibody diagnostic test (as such not masked by treatment status) rather than a disease activity biomarker. However, the clinical data to address the question of whether prevalence of seropositivity increases in severe asthma as a function of duration of disease were not collected.

The diffuse cytoplasmic staining of eosinophils with HC/patient serum and FITC-labelled anti-IgG and the high prevalence of serum IgG to eosinophil proteins such as EPX in both serum from HC s and patients suggest that serum anti-eosinophil autoantibodies may potentially be common in both health and disease. A limitation of our experiments is that we only looked for IgG autoantibodies, and not IgA/IgM/IgE, and could not determine the protein epitope to which the autoantibodies may bind or the IgG subclass. Elevated IgG4 subclass immunoglobulin can in particular be a feature of EGPA (42). Potentially, differences in these characteristics of any anti-eosinophil protein antibodies may determine whether they have pathological action. A number of our asthma patients had positive anti-MPO antibodies in our experimental assays, and clinical ANCA results, but did not have other aspects of EGPA to support a diagnosis of the systemic, vasculitic autoimmune disease. Potentially, the epitope for anti-MPO antibodies is different in these patients to those with EGPA (43).

However, autoantibodies within the pulmonary compartment, as detected in sputum, may be more important than autoantibodies in serum (44, 45). In particular, autoantibodies to eosinophil peroxidase in sputum is a feature of severe eosinophilic asthma and not apparent in sputum from HC s (6). Possibly, it is not the development of autoantibodies to eosinophil proteins that is abnormal in severe asthma, but local production of the autoantibody in the lungs is the issue. This is consistent with the published negative association between peripheral blood lymphocyte counts and sputum autoantibodies, suggesting that migration of B lymphocytes to inflamed lungs is a determinant of airway autoimmune responses (7).

In this research, we focussed on potential autoantibodies to eosinophils with the hypothesis that the clinical effectiveness of eosinophil-suppressing anti-IL-5 therapies may in part be the reduction of a circulating eosinophil autoantigen. Since this research was initiated, sputum autoantibody responses to macrophage receptor with collagenous structure (MARCO) have recently been reported in severe asthma (7, 46), challenging the criticality of eosinophils in asthma immunology. Importantly, IL-5 has actions on cell types other than eosinophils, including B cells (47, 48), and the effects of anti-IL-5 biologics to block IL- 5-dependent actions on these cell types may be of underappreciated importance.

In conclusion, although we did not find serum autoantibody responses to oxPTM proteins to be frequent in severe eosinophilic asthma and EGPA, we did find high proportions of patients to have autoantibodies to TREM1 and to PMA-stimulated neutrophils undergoing NETosis. It is increasingly apparent that severe asthma is a heterogeneous condition and different endotypes may be associated with different autoantibodies. As such, a serological diagnostic test may require a panel of autoantigens rather than a single antigen, as has been reported in other diseases (49, 50), and consistent with that, we found that the proportion of participants positive to one or more of ColV, MPO, TREM1, and IL1R2 was higher in patients with severe eosinophilic asthma than in HC s.

## Data availability statement

The raw data supporting the conclusions of this article will be made available by the authors, without undue reservation.

## Ethics statement

The studies involving human participants were reviewed and approved by London - Central Research Ethics Committee (NHS REC). The patients/participants provided their written informed consent to participate in this study.

## Author contributions

The research was conceived by PP, ML, and AN. IE and IK conducted the majority of experiments. MS and RB supported specific experimental methodologies. PP, ML, CG, SM-S, JB, RS, and GV contributed to patient recruitment and data collection and to translation of immunology findings to clinical pathology. All authors contributed to and reviewed this manuscript. All authors contributed to the article and approved the submitted version
