# Supplementary material for: Identification of autoantigens and their potential post-translational modification in EGPA and severe eosinophilic asthma
Source: Front Immunol. 2023 Jun 2;14:1164941. doi: 10.3389/fimmu.2023.1164941 (PMC10272393; doi:10.3389/fimmu.2023.1164941)
Supplement: Supplementary file 1 [file DataSheet_1.pdf]

Supplemental Information

Supplemental Methods

Proteomics analysis of the EPX protein

To test *in-vitro* acute oxidation of the Eosinophil Peroxidase Protein (EPX), 20 µg of commercially available EPX protein was divided in 5 µg aliquots. One aliquot was labelled as native and not subjected to *in-vitro* oxidation, whereas the other aliquots were exposed separately to increasing concentrations (0.2, 0.4 and 0.8 mM) of sodium hypoclorite (NaOCl), the oxidising agents. Prior to mass spectrometry analysis the EPX protein samples (treated and untreated) were column purified using Zeba™ Spin Desalting Columns (7K MWCO) (ThermoFisher), to remove the sodium hypochlorite from the NaOCl-treated samples, and to buffer-exchange all the samples in 50 mM ammonium bicarbonate solution (pH 8.0).

Immediately prior to proteomics analysis, the EPX protein samples were denatured in ProteaseMAX™ Surfactant (Promega), oxidised thiols were reduced with DTT, and subsequently alkylated using iodoacetamide. Trypsin Gold (Promega) endopeptidase was added at ratio 1:20 w/w enzyme: protein, and incubated for 3 hrs at 37 °C.

Ultra-performance liquid chromatography coupled with an electrospray ionization quadrupole time-of flight mass spectrometry operating in MSE mode (UPLC-qTof/MSe) was used to identify all Tryptic peptides from NaOCl-treated and untreated EPX protein samples, and to generate fragment ions upon collision induced dissociation (CID) to positively confirm their sequences. The analysis was executed on the ACQUITY H-Class UPLC system (Waters, Milford, USA) coupled to a qTOF High Definition Mass Spectrometer (HDMS) Synapt G2Si, equipped with an electrospray ionization (ESI) interface (Waters, Milford, USA). Tryptic peptides were separated using a Waters Acquity UPLC BEH C18 column (1.7 µm, 2.1 mm × 50 mm, and mobile phases consisted LCMS-grade water, 0.1% formic acid, and LCMS-grade Acetonitrile, 0.1% formic acid. MS data were collected from m/z 100–1500 Da in positive MSE continuum mode.

The electrospray ionization conditions were set as follows: capillary voltage, 3.5 kV; cone voltage, 40 V; cone gas flow, 50 L/h; source temperature, 120°C; desolvation gas flow, 800 L/h; and desolvation temperature, 450°C. Collision energy was set at 4 V in low energy acquisition; whereas high energy collision energy ramp wat set at 10–40 V. Leucine Encephalin (m/z 556.2771) was used for lock mass at a concentration of 200 pg/mL and a flow rate of 20 µL/min.

Data acquisition was carried out with Masslynx v4.2 (Waters, Milford, USA), whereas data was processed using UNIFI Scientific Information System v1.8 software (Waters, Milford, USA).

Supplemental Tables

ELISA	ColV	MPO	TREM1	IL1R2	EPX
Results	92	97	119	119	118
No sample available	23	21	1	1	0
Sample exclusion for technical reason	5 (high background OD for individual negative control)	2 (aberrantly high variability between replicates for samples)	0	0	2 (plating error)
TOTAL	120	120	120	120	120

Table S1: Numbers of participant samples unavailable for individual ELISAs

	Neutrophil Immunofluorescence	Eosinophil Immunofluorescence
**Number of Participants**	29	11
**Gender**		
**Female**	16	9
**Male**	13	2
**Age (mean)**	47.9	48.1
**Disease**		
**Healthy Control**	7	5
**Severe Eosinophilic Asthma**	17	1
**EGPA**	5	2
**Other**	0	3
**Treatment status**		
**On long-term oral corticosteroid**	12	4
**On biologic**	16	0
**On steroid-sparing immunosuppressant**	5	3

Table S2: Participant Characteristics for Neutrophil and Eosinophil Immunofluorescence Experiments
Numbers of participants except Age (mean years). EGPA; eosinophilic granulomatosis with polyangiitis. MA; moderate asthma. GPA/AAV; granulomatosis with polyangiitis or ANCA-associated vasculitis other than EGPA. COPD; chronic obstructive pulmonary disease. N/A; not applicable.

Supplemental Figures

Native EPX, tryptic fragment 82-83, AA546-AA550

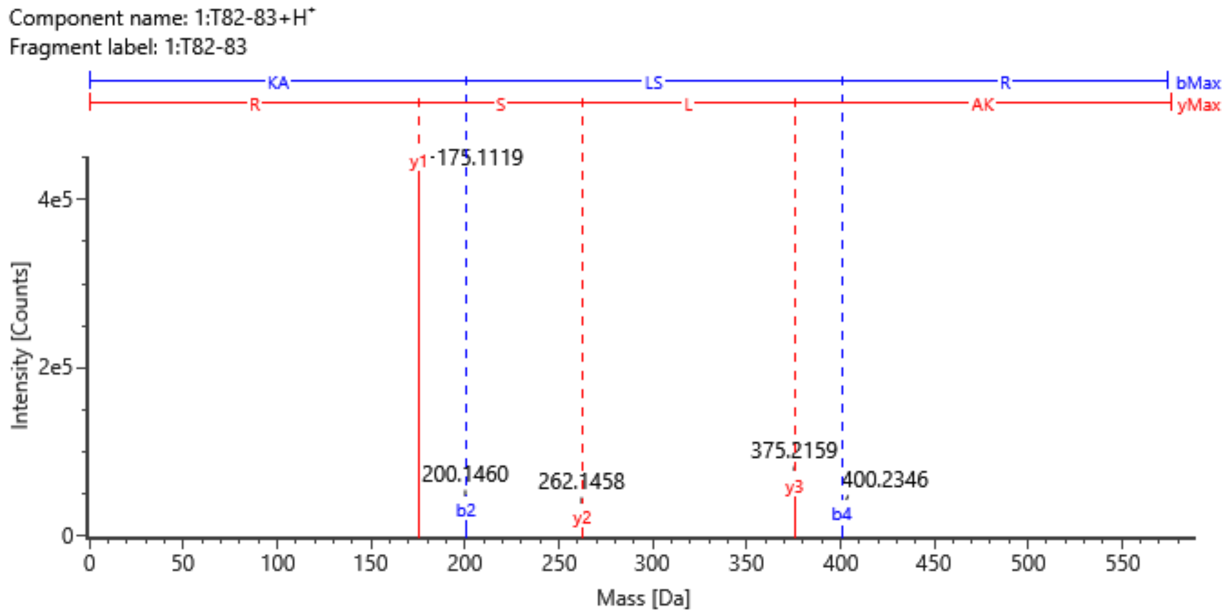

Figure S1: Fragmentation of protonated tryptic peptide KALSR (AA546-550) (m/z 574.3671) of the native EPX protein. Embedded **Table** displays all the expected b and y fragments, whereas the observed b and y fragments ions, with their corresponding m/z values, are marked in either blue (b ions) or red (y ions).

1A. Precursor m/z 574.3671 (yMax)

b1	129.1	**K**		
**b2**	**200.1**	**A**	446.3	y4
b3	313.2	**L**	**375.2**	**y3**
**b4**	**400.3**	**S**	**262.2**	**y2**
		**R**	**175.1**	**y1**

Modified EPX, Chloroamine (K,R), tryptic fragment 82-83, AA546-AA550

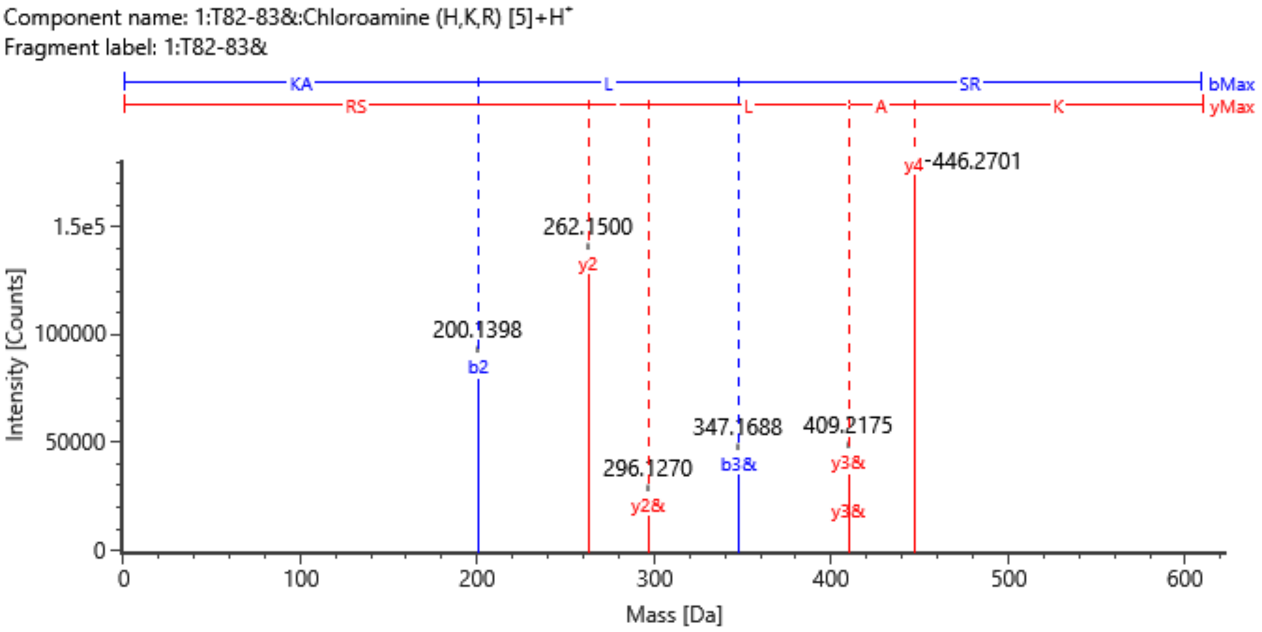

Figure S2: Fragmentation of the chloroamine-modified protonated tryptic peptide KALSR (AA546-550) (m/z 608.3271) of the EPX-treated protein (400 uM of oxidizing HOCL). The mass spectrum displays fragment ions for the lysine and arginine chloroamine-modified residues (K* and R*) (+33.9610 amu). Embedded **Tables 2A** and **2B** display all expected b and y fragments, whereas the observed b and y fragments ions, with their corresponding m/z values, are marked in either blue (b ions) or red (y ions).

Table 2A. List of all fragments expected from the chloroamine-modified lysine residue in the tryptic peptide. The observed fragments in either red or blue confirm the modification; expected ion b3, m/z 313.2 versus observed b3 ion m/z 347.2, confirms modification (+33.9610 amu). Similarly for expected and observed y4 and y2 ions.

Table 2B. List of all fragments expected from the chloroamine-modified Arginine (R*) residue in the tryptic peptide. The observed fragments in either red or blue confirm the modification; expected ion y3, m/z 375.2 versus observed y3 ion, m/z 409.2, confirms modification (+33.9610 amu). Similarly for expected and observed y2.

2A. Precursor m/z 608.3271 (yMax)

b1	163.1	K*		
**b2**	**200.1**	**A**	**446.3**	**y4**
**b3**	**347.2**	L	409.2	y3
b4	434.2	**S**	**262.2**	**y2**
		R	175.1	y1

2B.Precursor m/z 608.3271 (yMax)

b1	129.1	K		
**b2**	**200.1**	A	480.2	y4
b3	313.2	**L**	**409.2**	**y3**
b4	400.3	**S**	**296.1**	**y2**
		R*	209.1	y1

Native EPX, tryptic fragment 49, AA329-AA334

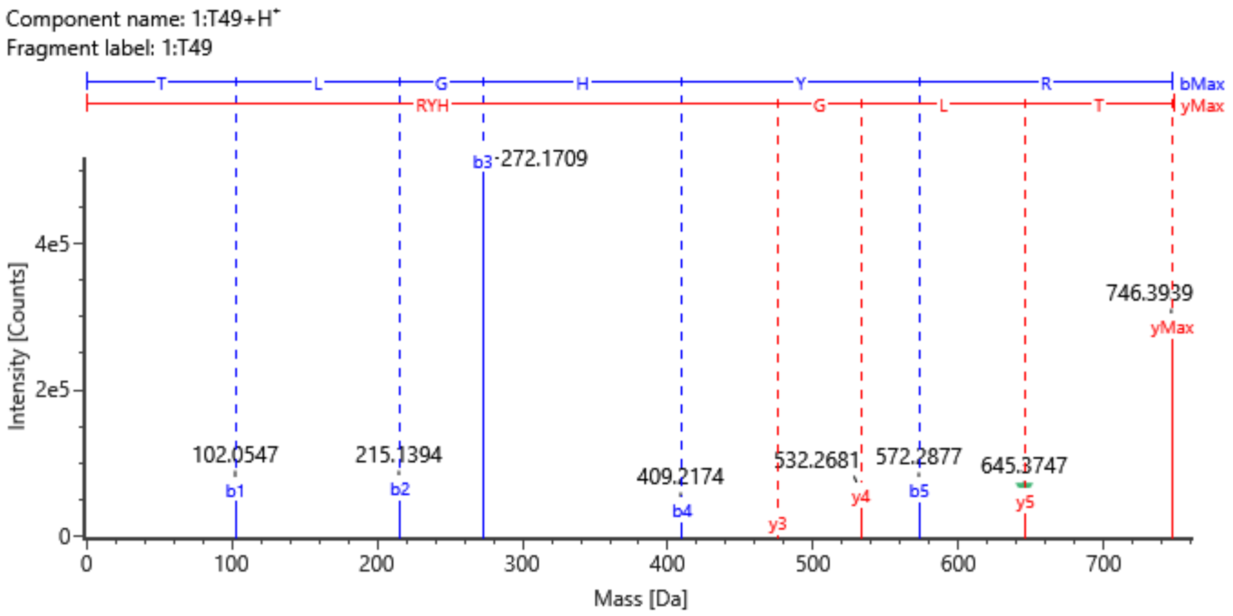

Figure S3: Fragmentation of the protonated tryptic peptide TLGHYR (AA329-334) (m/z 746.3944) of the native EPX protein. **Table 3A** displays all the expected b and y fragments, whereas the observed b and y fragments ions, with their corresponding m/z values, are marked in either blue (b ions) or red (y ions).

3A. Precursor m/z 746.3944 (yMax)

b1	102.1	T		
b2	215.1	L	645.4	y5
b3	272.2	G	532.3	y4
b4	409.2	H	475.2	y3
b5	572.3	Y	338.2	y2
		R	175.1	y1

Modified EPX, Tyr-DOPA(Y), tryptic fragment 48-49, AA328-AA334

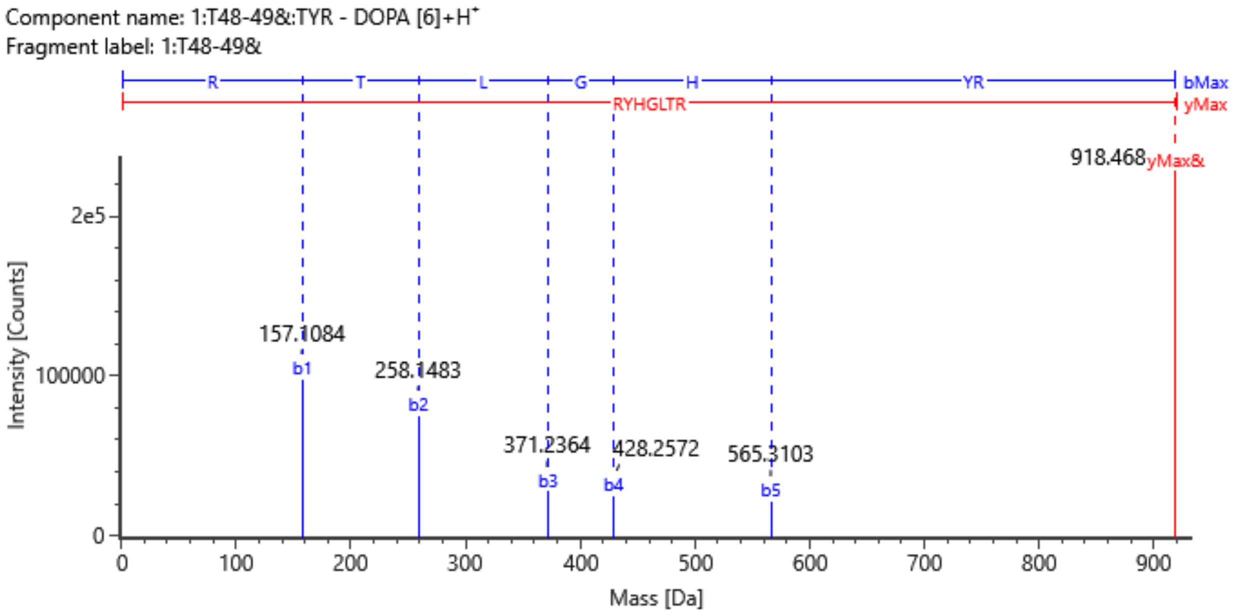

Figure S4: Fragmentation of the hydroxylated Tyrosine-modified protonated tryptic peptide TLGHYR (AA329-334) (m/z 918.4955) of the EPX-treated protein (400 uM of oxidizing HOCL). The mass spectrum displays observed fragment ions for the Tyrosine-modified residue (Y*) (+15.9949 amu). **Table 4A** displays all the expected b fragments marked in blue, whereas none of the expected y ions were observed. The observed Ymax fragment (m/z 918.468) on the spectrum confirms the Tyrosine to DOPA modification as the expected ion Ymax, is m/z 901.477, versus observed Ymax ion m/z 918.468, confirms modification (+15.9949 amu).

4A. Precursor m/z 918.4955 (yMax)

**b1**	**157.1**	**R**		
**b2**	**258.2**	**T**	762.4	y6
**b3**	**371.2**	**L**	661.4	y5
**b4**	**428.3**	**G**	548.3	y4
**b5**	**565.3**	**H**	491.2	y3
b6	744.38	**Y**	354.2	y2
		**R**	175.1	y1

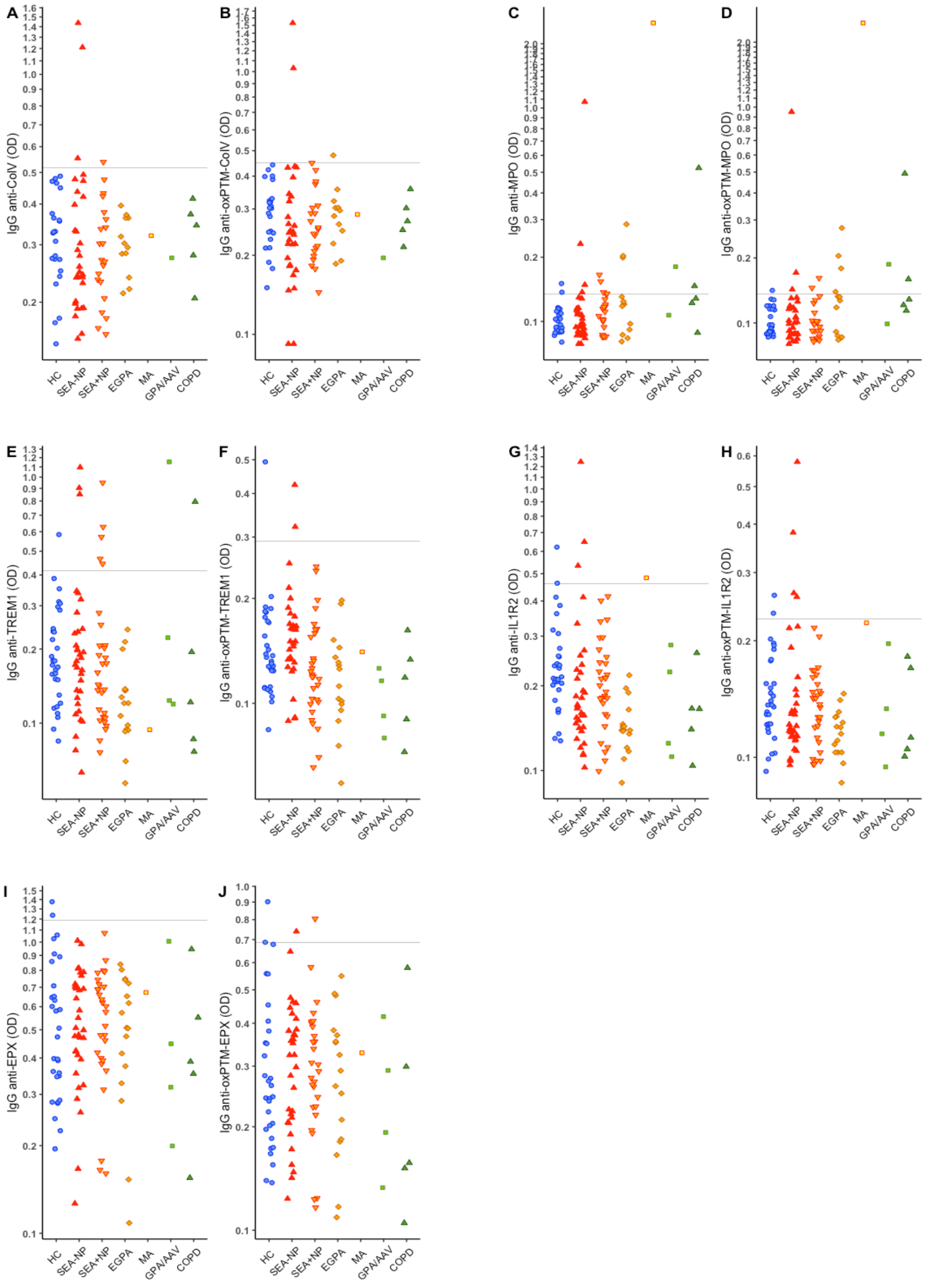

Figure S5: Serum autoantibodies to target native and oxidative post-translationally modified (oxPTM) proteins as measured by indirect ELISA across all participant groups.

ELISA optical density (OD) on a logarithmic scale (*y*-axis) as measure of serum IgG autoantibodies to target proteins in native and oxidative post-translationally modified form (oxPTM) in different participant groups (*x*-axis). HC; healthy control participants. SEA-NP; severe eosinophilic asthma without nasal polyps; SEA+NP; severe eosinophilic asthma with nasal polyps. EGPA; eosinophilic granulomatous polyangiitis. MA; moderate asthma. GPA/AAV; granulomatosis with polyangiitis or ANCA-associated vasculitis other than EGPA. COPD; chronic obstructive pulmonary disease. *y*-axis intercept at the upper limit of normal (ULN) for healthy control participant ELISA OD.

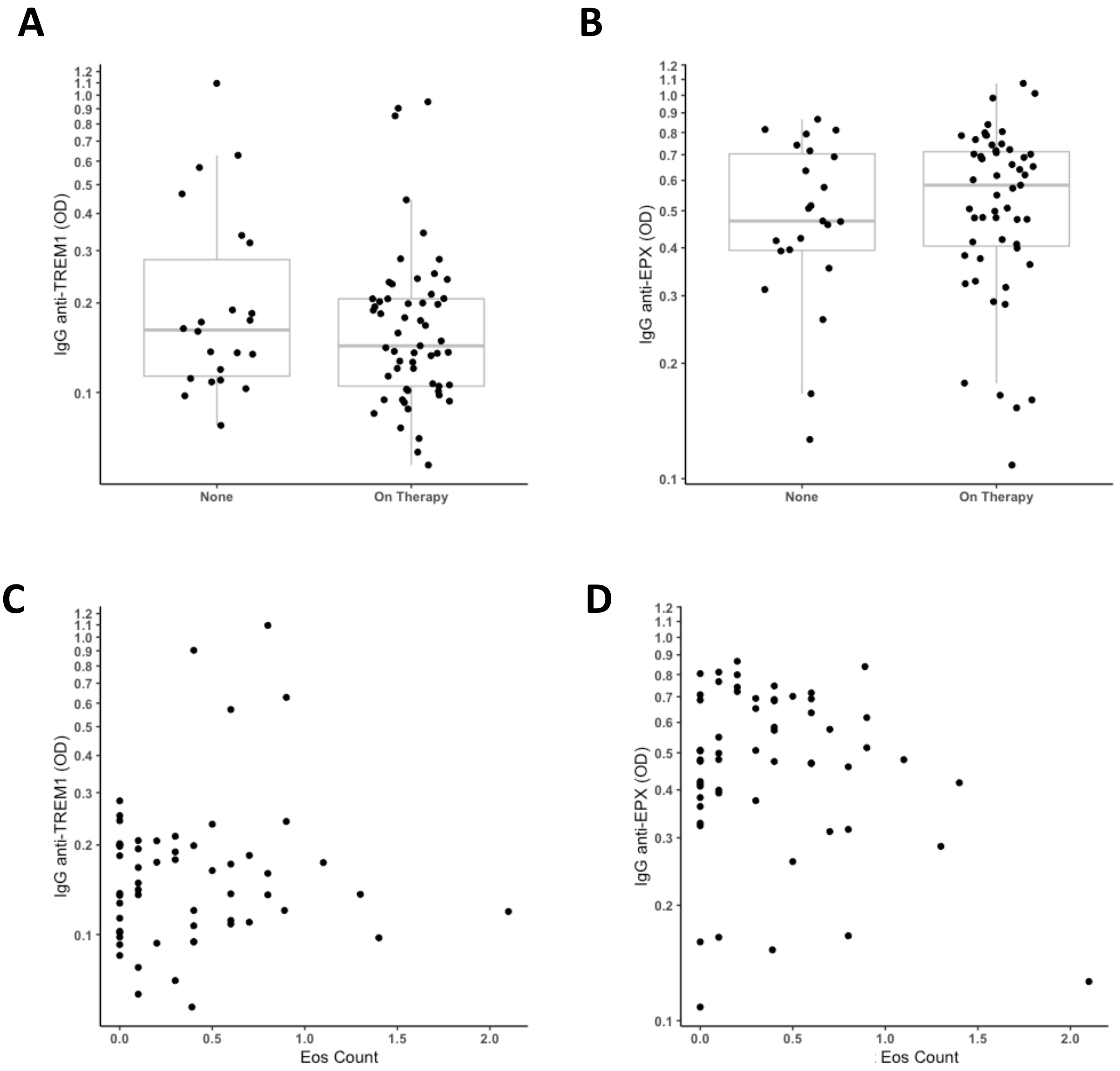

Figure S6: Further analyses of serum autoantibody ELISA ODs relative to clinical characteristics for patients with severe asthma and EGPA.

ELISA optical density (OD) on a logarithmic scale (*y*-axis) as measure of serum IgG autoantibodies to target proteins TREM1 (A,C) and EPX (B,D). **(A-B)** Patients stratified by whether at time of sample collection they were on systemic steroid-sparing immunosuppressant (e.g. azathioprine) / biologic therapy ("On Therapy") or not ("None"). **(C-D)** ELISA OD compared to most recent blood eosinophil count x10^9/L (taken within three months of date of research sample collection).
